# Case Report and Genetic Sequence Analysis of *Candidatus* Borrelia kalaharica, Southern Africa

**DOI:** 10.3201/eid2409.171381

**Published:** 2018-09

**Authors:** Katarina Stete, Siegbert Rieg, Gabriele Margos, Georg Häcker, Dirk Wagner, Winfried V. Kern, Volker Fingerle

**Affiliations:** Medical Center—University of Freiburg Faculty of Medicine, Freiburg, Germany (K. Stete, S. Rieg, G. Häcker, D. Wagner, W.V. Kern);; National Reference Center for Borrelia, Oberschleißheim, Germany (G. Margos, V. Fingerle)

**Keywords:** relapsing fever, *Borrelia*, bacteria, zoonoses, tickborne diseases, vector-borne infections, travel, Germany, southern Africa

## Abstract

Tickborne relapsing fever caused by *Borrelia* species is rarely reported in travelers returning from Africa. We report a case of a 71-year-old woman who sought treatment at University Medical Center in Freiburg, Germany, in 2015 with recurrent fever after traveling to southern Africa. We detected spirochetes in Giemsa-stained blood smears. Treatment with doxycycline for suspected tickborne relapsing fever was successful. Sequence analyses of several loci (16S rRNA, flagellin, *uvrA*) showed high similarity to the recently described *Candidatus* Borrelia kalaharica, which was found in a traveler returning from the same region earlier that year. We provide additional information regarding the genetic relationship of *Candidatus* B. kalaharica. Sequence information for an additional 6 housekeeping genes enables improved comparability to other borrelial species that cause relapsing fever. Our report underlines the importance and possible emergence of the only recently delineated pathogen in southern Africa.

An infection with *Borrelia* species bacteria causes relapsing fever (RF). It is transmitted by several arthropods, and dependent on the transmitting vector; louseborne relapsing fever (LBRF) is different from tickborne relapsing fever (TBRF) (*1**,**2*). The clinical picture of RF includes recurrent fever episodes accompanied by headache, hepatomegaly, splenomegaly, vomiting, conjunctivitis, myalgia, and arthralgia. It may be difficult to differentiate RF from other febrile illnesses, especially malaria. RF can be diagnosed by detection of spirochetes in blood smears or by PCR of EDTA-blood, and treatment is typically with penicillins or tetracyclines (*1**,**3*).

Whereas *B. recurrentis* is the cause of LBRF, which occurs mainly in the Horn of Africa, several *Borrelia* species may cause TBRF, which is found in many areas of the world. The endemic *Borrelia* species differ across geographic regions, and they have traditionally been divided into Old World and New World *Borrelia*. So far, ≈15 *Borrelia* species have been described to cause TBRF in humans worldwide (*1*). In Africa, TBRF has been traditionally attributed to *B. crocidurae* in western Africa, *B. hispanica* in northern Africa, and *B. duttonii* in eastern Africa (*1**,**4*).

Because microscopy is currently the standard method for diagnosis of TBRF in most countries in Africa, diagnosis does not usually include differentiation of species. With the advent of molecular diagnostic methods, scientists can identify species by sequencing different loci of *Borrelia* DNA from blood, such as the 16S rRNA gene, the flagellin gene (*flaB*), or the *glpQ* gene (*5**,**6*). Sequence analysis has challenged the assumption of strict division of species across Africa not only by the detection of geographic overlap of several *Borrelia* species, but also by detection of previously unknown species (*6*). Moreover, a *Borrelia* species found in ticks and in human blood in Tanzania showed more homology to New World *Borrelia* species than to the species known to be present in Africa (*7**–**9*). These findings were based on 16S rRNA and *flaB* partial sequences, which were deposited in GenBank as *B. duttonii* (accession nos. AB113315, AB105169, AB105132, AB057547, and AB105118). In 2015, a case of RF was described in a German tourist after traveling to the Kalahari Desert. The strain also showed greater genetic homology to New World *Borrelia* spp. and was proposed as a new species *Candidatus* B. kalaharica on the basis of the analysis of 16S rRNA, *flaB,* and *uvrA* genes (*10*)*.*

Although RF is believed to be endemic to many areas in Africa, it is rarely diagnosed in travelers returning from these regions (*11*). In previous years, several cases of LBRF have been reported from several countries in Europe in migrants from eastern Africa (*2**,**3**,**12**–**16*). Reports on TBRF in travelers returning from Africa to Europe are limited to case reports. Most of these infections were acquired in West Africa (*17**–**23*), with single reports from other areas, such as Ethiopia and Morocco (*21**,**24*).

We present a case of TBRF in a tourist from Germany returning from southern Africa and describe the results of a comprehensive molecular diagnostic analysis that underlines that *Candidatus* B. kalaharica represents a new species that is genetically distant from other RF group species and that it appears to be an emerging pathogen for humans that should be considered in the differential diagnosis of febrile patients. We obtained written informed consent from the patient for publication.

## Materials and Methods

We performed slide microscopy after standard Giemsa staining of a thick and a thin blood smear. We obtained photographs from a 100× magnification objective using a Nikon Eclipse Ni microscope (Nikon Corporation, Tokyo, Japan).

We initiated in vitro cultures of infected blood using medium and conditions as previously described for RF species (*25**,**26*). We performed DNA extraction from EDTA blood using the Maxwell 16 FFS Nucleic Acid Extraction System Custom Kit (Promega, Mannheim, Germany) according to the manufacturer’s instructions. We amplified fragments of the 16S rRNA, *glpQ* and *flaB* using primers and PCR conditions as described previously (*25**,**27**,**28*). We performed multilocus sequence analysis (MLSA) on housekeeping genes (*clpA, clpX, nifS, pepX, pyrG, rplB, recG,* and *uvrA*) as described (*29*; Technical Appendix Table 1). For PCR we ran HotStarTaq Mastermix (QIAGEN, Hilden, Germany) as touch-down protocol for the first 9 cycles with annealing temperatures of 55°C–48°C, decreasing 1°C each cycle, followed by 32 cycles at 48°C annealing temperature. The temperature profile was 95°C for 15 min for activation of Taq polymerase, 94°C for 30 s for denaturation, 30 s for annealing at the temperatures given previously, and 72°C for 60 s for elongation. A final step of elongation was at 72°C for 5 min, and then we held the samples at 12°C.

We used GATC Biotech AG (Konstanz, Germany) for sequencing, and performed sequence alignment, genetic distance analyses, and construction of phylogenetic trees in MEGA5 (*30**,**31*). We used BLAST (*32*) to compare the sequences we obtained (GenBank accession nos. KY560340–8) to sequences in GenBank (accession numbers in Technical Appendix Tables 2, 4) using standard settings. We conducted genetic distance analyses in MEGA5 (*31*) using the Kimura 2-parameter model (*30*). We inferred the evolutionary history by using the maximum likelihood method based on the general time-reversible model (*33*). We generated the initial trees for the heuristic search automatically by applying neighbor-joining and BioNJ algorithms to a matrix of pairwise distances estimated using the maximum composite likelihood approach, and then selecting the topology with superior log likelihood value. We calculated node support values with 1,000 bootstrap repeats. We used discrete gamma distribution to model evolutionary rate differences among sites [+G]. The rate variation model allowed for some sites to be evolutionarily invariable [+I]. The trees are drawn to scale, with branch lengths measured in the number of substitutions per site. Codon positions included were 1st + 2nd + 3rd + noncoding for *flaB* sequences and housekeeping gene sequences. We eliminated all positions containing gaps and missing data.

## Results

### Case Report

A 71-year old woman sought treatment for fever after a 4-week camping trip to South Africa, Namibia, Botswana, and Zimbabwe. The patient reported no malaria chemoprophylaxis, fresh water contact, or tick bites. Other than horseback riding, she could recall no direct contact with animals. Preexisting conditions were nonmetastatic breast cancer under treatment with exemestan and a history of penicillin allergy.

**Figure 1:**
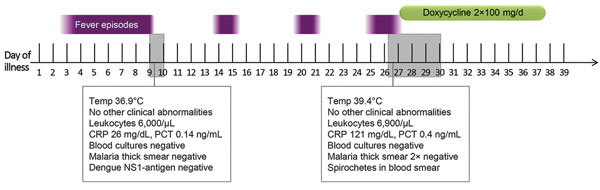
Timeline of the course of symptoms and treatment, including laboratory test results, for a patient with recurrent fever after traveling to southern Africa, 2015. Temp, temperature; CRP, C-reactive protein; PCT, procalcitonin.

The patient reported fever episodes starting 3 days before returning to Germany. Malaria was ruled out at a local health unit in South Africa by thick smear microscopy. Three days after arriving in Germany, the patient came to our clinic with a history of fever but no other abnormal signs or symptoms. Leukocyte counts were normal; levels of C-reactive protein and procalcitonin were slightly elevated (Figure 1). A malaria thick blood smear, blood cultures, and a dengue nonstructural protein 1 antigen test showed negative results. The fever resolved spontaneously, and the patient was discharged and asked to return in case of recurrence of symptoms.

**Figure 2:**
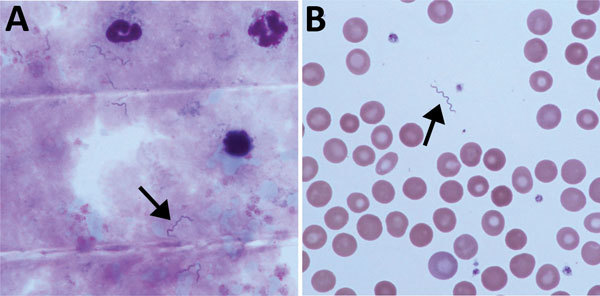
Microscopy of blood from a patient with recurrent fever episodes after traveling to southern Africa, 2015. Arrows indicate spirochetes. A) Thick smear specimen; B) thin smear specimen. Original magnification 100×.

Seventeen days later, the patient returned with RF (temperature >39°C). She reported 2 episodes of fever lasting 2–3 days flanked by symptom-free intervals of ≈4 days (Figure 1). Leukocyte counts again were normal; and levels of C-reactive protein and procalcitonin were elevated. We detected no malaria parasites in a thick smear; however, we found multiple spirochetes compatible with *Borrelia* species (Figure 2). We made a presumptive diagnosis of TBRF on the basis of the travel route and with no evidence of body lice infestation. We started antimicrobial therapy with doxycycline (2 × 100 mg/d) and close monitoring. We observed no signs of a Herxheimer reaction. PCR diagnostics of 16S rRNA confirmed the diagnosis of *Borrelia* infection. For further species differentiation, we sent a blood sample to the German National Reference Center for sequence analysis for *Borrelia.* An 11-day course of doxycycline led to an uneventful recovery with no recurrence of fever.

### Sequence Analysis and Phylogeny

To investigate the *Borrelia* species designation, we conducted BLAST searches using the 16S rRNA PCR fragment. Top hits included *Candidatus* B. kalaharica, *B. duttonii* strain VS4, *B. turicatae,* and *B. parkeri*. Genetic distance analyses using the 16S rRNA fragments in MEGA5 (*31*) revealed strains *Candidatus* B. kalaharica (*10*) and VS4 from Tanzania, an atypical RF strain present in the Old World (*8*), as closest matches (Technical Appendix Table 2). Although designated *B. duttonii* in GenBank, VS4 was closely related to some strains found in the Mvumi region of Tanzania (*7*) which were shown to be more closely related to New World RF species than to *B. duttonii*. Genetic distance values obtained for the 16S rRNA fragment were 0.2% for *Candidatus* B. kalaharica and slightly higher for *B. parkeri*, *B. crocidurae*, and *B. turicatae* (0.4%) (Technical Appendix Table 2).

When the sequence of a flagellin gene (*flaB*) fragment (252 bp) was used for genetic distance analysis, *Candidatus* B. kalaharica was again the most closely related strain, with genetic distance value = 0.000 (Technical Appendix Table 3). Strains representing atypical *B. duttonii* (*7**,**8*) showed higher genetic distance values (strain TnB, 0.8%; strain EM14, 1.2%), whereas for other *Borrelia* species such as *B. anserina* BA2 (5.8%), *B. turicatae* (6.2%) and *B. parkeri* (6.2%), the values were even higher, indicating a close genetic relationship of the strain investigated here to *Ca*. B. kalaharica. This was also reflected in phylogenies (Technical Appendix Figures 1, 2). In the 16S rRNA phylogeny, the DNA isolate investigated here formed a clade together with *Candidatus* B. kalaharica and VS4 from Mvumi, Tanzania (*8**,**10*). In the *flaB* phylogeny, our DNA isolate and *Candidatus* B. kalaharica formed a sister clade to strains from the Mvumi region in Tanzania (*7**,**8*), notably outside the clade containing Old World RF species such as *B. duttonii*, suggesting that they are divergent from *B. duttonii*.

**Figure 3:**
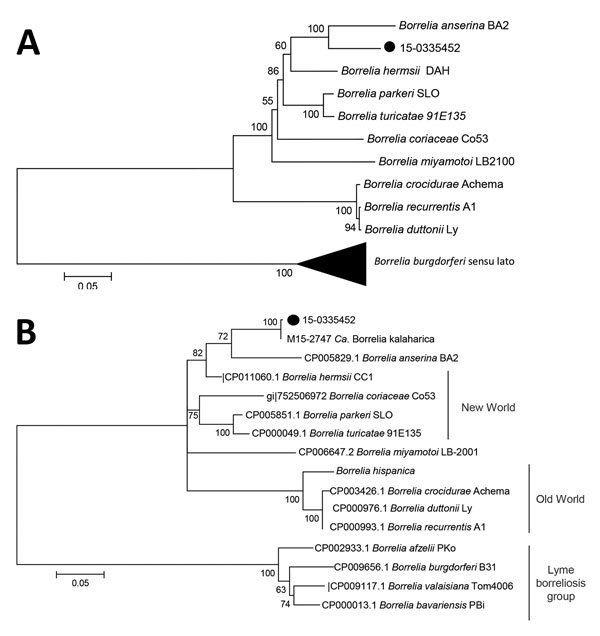
Molecular phylogenetic analysis by maximum-likelihood method of isolates from a patient in Germany with recurrent fever episodes after traveling to southern Africa, 2015. A) Phylogeny of *uvrA* sequence fragments. The tree with the highest log likelihood (–2566.8936) is shown. A discrete gamma distribution was used to model evolutionary rate differences among sites (4 categories [+G, parameter = 0.9541]). The rate variation model allowed for some sites to be evolutionarily invariable ([+I], 43.5691% sites). The tree is drawn to scale, with branch lengths measured in the number of substitutions per site. The analysis involved 19 nt sequences. There were a total of 570 positions in the final dataset. Bootstrap values >50 are shown. Black dot indicates the sample analyzed in this study. Black triangle represents the clade containing *B. burgdorferi* s.l. isolates, collapsed for simplicity. Scale bar indicates substitutions per site. B) Phylogeny of concatenated sequences of 7 MLST housekeeping loci (*clpX, nifS, pepX, pyrG, recG, rplB, uvrA*). The tree with the highest log likelihood (−31066.7852) is shown. The percentage of trees in which the associated taxa clustered together is shown next to the branches. A discrete gamma distribution was used to model evolutionary rate differences among sites (4 categories [+G, parameter = 0.7881]). The rate variation model allowed for some sites to be evolutionarily invariable ([+I], 36.6955% sites). The analysis involved 33 nt sequences. There were a total of 4,203 positions in the final dataset. The subtree containing the LB group of spirochetes was collapsed. Bootstrap values >50 are shown. Black dot indicates the sample analyzed in this study. Scale bar indicates substitutions per site.

We obtained similar results using 7 housekeeping loci (Figure 3; Technical Appendix Tables 4, 5) and, in particular locus *uvrA.* For this locus, sequences of *Candidatus* B. kalaharica were available (Figure 3, panel A). Genetic distance analysis (Technical Appendix Tables 4, 5) and phylogenetic inferences (Figure 3, panel B) further support the close genetic relationship of the specimen investigated here with *Ca*. B. kalaharica and both clusters next to *B. anserina*. For MLST analysis, only 7 genes could be included as *clpA* PCR did not yield a PCR product. The PCR for the *glpQ* locus also proved negative in spite of several amplification attempts suggesting that perhaps base differences in the primer regions prevented amplification. Despite our efforts, we were unable to cultivate the causative pathogen from blood.

## Discussion

The case described here is the second report within a few months of TBRF in a tourist from Germany traveling to countries in southern Africa, such as South Africa, Namibia, and Botswana (*10*). In the previous case, a presumed soft tick bite in the Kalahari Desert was described, whereas our patient did not report any arthropod bite. However, contact with arthropods was likely as the patient was camping. Soft tick *Ornithodoros* species only need short blood meals and do not attach tightly to the host (*34*), making it conceivable that a feeding tick was not noticed. These cases underscore that, in returning travelers with RF, TBRF should be considered in the differential diagnosis, even if no tick bite is reported. Thick smears are the diagnostic procedure of choice and should be carefully evaluated for corkscrew-shaped spirochetes (*1*). However, the sensitivity of this method may change depending on febrile versus afebrile periods with different pathogen loads circulating in the blood. Thus, as we saw in this patient, thick smears may turn negative during infection and should therefore be repeated preferentially during febrile episodes.

Death as a result of TBRF is considered to be rare; however, higher mortality rates have been suspected as a result of Herxheimer reactions, even though there is a lack of data for TBRF in Africa (*1**,**35*). Clinicians need to be aware that the initiation of antimicrobial treatment might be associated with a severe Herxheimer reaction, necessitating aggressive supportive care.

*Borrelia* species can be identified and differentiated by means of DNA sequence analysis, although it may be hard to distinguish closely related *Borrelia* species, such as *B. duttonii*, *B. recurrentis,* and *B. crocidurae* (*36**,**37*)*.* 16S rRNA sequences are available for many of the *Borrelia* species and strains that have been found in Africa and thus, although the locus may have low resolution, it can give a first indication of relationships. Other loci that have been used in previous reports were also used in the current study, including *flaB* and housekeeping loci (*37*)*.* Because there is so little information about which RF-causing species do occur in southern Africa (*6*), a more thorough characterization of the DNA isolate would be beneficial to epidemiologists and other researchers in the field.

The traditional concept of strict division of geographic areas into Old World and New World *Borrelia* and division of species across Africa has been challenged by the description of new *Borrelia* species. This is the second report of a species that has not been described previously. Genetically, *Candidatus* B. kalaharica is most closely related to TBRF *Borrelia* described from the Mvumi region in Tanzania (*8**,**38*). In previous publications it was suggested that these *Borrelia* strains from Mvumi may belong to the new species (*8**,**38*). Unfortunately, the only available sequences were for 16S rRNA and *flaB,* but more sequence data will be needed to reveal the taxonomic position of these strains. Of interest, both the strains from Mvumi and *Candidatus* B. kalaharica show more genetic similarity to New World RF species than to the expected Old World species.

We report the second case of a human infection with the proposed new species *Candidatus* B. kalaharica. Our findings support the definition of *Candidatus* B. kalaharica as a new species that is genetically distant from other RF group species and more closely related to New World RF *Borreliae*. It appears to be an emerging pathogen for humans that should be considered in the differential diagnosis of febrile patients.

Technical Appendix Additional information about genetic analysis of *Borrelia* species and *Candidatus* Borrelia kalaharica
