# Supplementary material for: Case Report and Genetic Sequence Analysis of Candidatus Borrelia kalaharica, Southern Africa
Source: Emerg Infect Dis. 2018 Sep;24(9):1659–64. doi: 10.3201/eid2409.171381 (PMC6106409; doi:10.3201/eid2409.171381)
Supplement: Technical Appendix — Additional information about genetic analysis of Borrelia species and Candidatus Borrelia kalaharica [file 17-1381-Techapp-s1.pdf]

Article DOI: https://doi.org/10.3201/eid2409.171381

Case Report and Genetic Sequence Analysis of *Candidatus* Borrelia kalaharica, Southern Africa

Technical Appendix

Technical Appendix Table 1. Primers used for MLST of *Borrelia* spp. housekeeping loci *clpA, clpX, nifS, pepX, pyrG*, recG, *rplB,* and *uvrA**

Primer designation	Primer sequence 5′ →3′	Used for	Result
clpAF1262	AAGCTTTTGAYYTATTAGATGGTC	PCR	No PCR product
clpAR2276	TCATATTTDATRGTDTCGTC	PCR	No PCR product
clpXF109	GCYATTTGTTTTGAATGTTCTAAAATATG	PCR/sequencing	Good sequence
clpXR1277	TAAAGTTCTTTTGCCCAAGG	PCR/sequencing	Sequencing failed
nifSFm23	TAAAAATAAAGAGTACTAAATAAATG	PCR/sequencing	Good sequence
nifSR899	CCAAGACCAATAATTCCTGC	PCR	Good PCR
nifS716	GGAGCAAGCATTTTYTGTC	Sequencing	Sequencing failed
pepFX361	AGAGAYTTAAGYTTAKCAGG	PCR	Good PCR
pepXF364	GAYTTAAGYTTAKCAGGARTTG	Sequencing	Sequencing failed
pepXR1207	CYATAGTTTCTCTTAAAGAYTGC	PCR	Good PCR
pepXR1187	TGCATTCCCCACATTGG	Sequencing	Good sequence
pyrGF379	TATTTAGGKAGAACTGTACAGC	PCR/sequencing	Sequencing failed
pyrGR1261	CAGCATCAAYTATRCCACAAAC	PCR	Good PCR
pyrG1252	TATRCCACAAACATTACGKGC	Sequencing	Good sequence
recGF898	GCKTTTCTMTCTAGYATTCC	PCR/sequencing	Good sequence
recGR1779	TTCRGTTAAAGGTTCCTTATAAAG	PCR/sequencing	Sequencing failed
rplBF3	GGAGAAAAATATGGGKATTAAGAC	PCR	Good PCR
rplBF18	ATTAAGACTTATARGCCAAAAAC	Sequencing	Good sequence
rplBR769	GRCCCCAAGGWGATAC	PCR	Good PCR
rplBR759	GATACAGGATGWCGACC	Sequencing	Sequencing failed
uvrAF1173	GCGTTATCTTWCAACTGAATC	Forward	Sequencing failed
uvrAR2153	CTAATCTCDGTAAAAAATCCAACATAAG	Reverse	Good sequence

*Source: https://pubmlst.org/borrelia/. *F,* forward primer; R, reverse primer; MLST, multilocus sequence typing..

Technical Appendix Table 2. Estimates of evolutionary divergence between 16S sequences in *Borrelia* spp. using the Kimura 2-parameter model*

No.	Sequence	1	2	3	4	5	6	7	8	9	10	11	12	13	14	15	16	17	18	19	20	21
1	15–0335452																					
2	*Candidatus* Borrelia kalaharica M15–2747	0.002																				
3	AB113315.1 *Borrelia duttonii* VS4	0.002	0.004																			
4	CP005851.1 *Borrelia parkeri* SLO	0.004	0.007	0.007																		
5	NR 102961.1 *Borrelia crocidurae* Achema	0.004	0.007	0.007	0.004																	
6	CP000976.1 *Borrelia duttonii* Ly	0.004	0.007	0.007	0.004	0.000																
7	CP000049.1 *Borrelia turicatae* 91E135	0.004	0.007	0.007	0.000	0.004	0.004															
8	CP011060.1 *Borrelia hermsii* CC1	0.011	0.013	0.013	0.007	0.011	0.011	0.007														
9	CP005829.1 *Borrelia anserina* BA2	0.018	0.020	0.020	0.018	0.023	0.023	0.018	0.016													
10	NR 121718.1 *Borrelia coriaceae* Co53	0.016	0.018	0.018	0.011	0.011	0.011	0.011	0.018	0.030												
11	JF803950.1 *Borrelia microti* IR-1 16S	0.007	0.009	0.009	0.007	0.002	0.002	0.007	0.013	0.025	0.013											
12	|U42294.1 *Borrelia hispanica*	0.007	0.009	0.009	0.007	0.002	0.002	0.007	0.013	0.025	0.013	0.004										
13	U42300.1 *Borrelia recurrentis*	0.009	0.011	0.011	0.009	0.004	0.004	0.009	0.016	0.027	0.016	0.007	0.007									
14	NR 024820.1 *Borrelia turcica* IST7	0.020	0.023	0.023	0.016	0.020	0.020	0.016	0.013	0.020	0.027	0.023	0.023	0.025								
15	AY682920.1 *Borrelia lonestari* MO2002-V1 1	0.023	0.025	0.025	0.018	0.023	0.023	0.018	0.020	0.032	0.016	0.025	0.023	0.027	0.029							
16	KF569941.1 *Borrelia theileri* KAT	0.018	0.020	0.020	0.013	0.018	0.018	0.013	0.016	0.027	0.007	0.020	0.020	0.023	0.025	0.009						
17	gi|890832120 *Borrelia miyamotoi* LB-2001	0.027	0.030	0.030	0.023	0.027	0.027	0.023	0.025	0.027	0.020	0.030	0.027	0.032	0.034	0.016	0.013					
18	CP009656.1 *Borrelia burgdorferi* ss B31	0.020	0.022	0.022	0.025	0.025	0.025	0.025	0.022	0.027	0.036	0.027	0.025	0.029	0.025	0.034	0.034	0.041				
19	CP009117.1 *Borrelia valaisiana* Tom4006	0.034	0.032	0.036	0.034	0.039	0.039	0.034	0.032	0.032	0.046	0.041	0.039	0.044	0.029	0.044	0.043	0.046	0.016			
20	CP002933.1 *Borrelia afzelii* PKo	0.034	0.032	0.036	0.034	0.039	0.039	0.034	0.032	0.027	0.046	0.041	0.039	0.044	0.034	0.044	0.043	0.041	0.016	0.009		
21	emb|X85193.1 *Borrelia garinii* DK27	0.027	0.029	0.029	0.027	0.032	0.032	0.027	0.025	0.029	0.029	0.034	0.032	0.036	0.027	0.027	0.027	0.034	0.009	0.016	0.016	
22	emb|X85199.1 *Borrelia bavariensis* PBi	0.034	0.036	0.036	0.034	0.039	0.039	0.034	0.032	0.036	0.036	0.041	0.039	0.044	0.034	0.034	0.034	0.041	0.016	0.018	0.018	0.007

*The number of base substitutions per site from between sequences are shown. The analysis involved 22 nt sequences. There were a total of 452 positions in the final dataset. Blank spaces indicate complete identity.

Technical Appendix Table 3. Estimates of evolutionary divergence between *Borrelia* spp. flagellin B (*flaB*) sequences*

No.	Sequence	2	3	4	5	6	7	8	9	10	11	12	13	14	15	16	17	18	19	20	21	22	23	24	25
2	15–335452																								
3	*Candidatus* Borrelia kalaharica M15–2747	0.000																							
4	AB057547.1 *Borrelia duttonii* TnB	0.008	0.008																						
5	AB105169.1 *Borrelia duttonii* EM14	0.012	0.012	0.004																					
6	CP005830.1 *Borrelia anserina* BA2	0.058	0.058	0.058	0.062																				
7	AB105119.1 *Borrelia duttonii* Tnf10	0.080	0.080	0.080	0.075	0.085																			
8	CP000049.1 *Borrelia turicatae* 91E135	0.062	0.062	0.062	0.067	0.071	0.084																		
9	CP005851.1 *Borrelia parkeri* SLO	0.062	0.062	0.062	0.067	0.080	0.093	0.016																	
10	JN402326.1 Uncultured *Borrelia* sp. clone AP174	0.067	0.067	0.067	0.071	0.076	0.089	0.016	0.024																
11	CP000993.1 *Borrelia recurrentis* A1	0.080	0.080	0.080	0.075	0.085	0.008	0.084	0.093	0.089															
12	CP000976.1 *Borrelia duttonii* Ly	0.080	0.080	0.080	0.075	0.085	0.000	0.084	0.093	0.089	0.008														
13	CP003426.1 *Borrelia crocidurae* Achema	0.080	0.080	0.080	0.075	0.085	0.008	0.084	0.093	0.089	0.008	0.008													
14	GU357612.1 *Borrelia hispanica* strain Sp3	0.084	0.084	0.084	0.080	0.089	0.012	0.089	0.098	0.093	0.020	0.012	0.020												
15	JF708951.1 *Borrelia microti* Abyek	0.084	0.084	0.084	0.080	0.080	0.004	0.089	0.098	0.093	0.012	0.004	0.012	0.016											
16	CP005745.1 *Borrelia coriaceae* Co53	0.093	0.093	0.097	0.093	0.097	0.111	0.084	0.084	0.089	0.121	0.111	0.121	0.116	0.116										
17	CP011060.1 *Borrelia hermsii* CC1	0.098	0.098	0.098	0.102	0.093	0.131	0.108	0.103	0.112	0.131	0.131	0.131	0.146	0.136	0.111									
18	KF569936.1 *Borrelia theileri* KAT	0.102	0.102	0.107	0.102	0.111	0.116	0.126	0.130	0.135	0.116	0.116	0.116	0.121	0.121	0.120	0.154								
19	AY850063.1 *Borrelia lonestari* MO2002-V1	0.112	0.112	0.121	0.117	0.121	0.145	0.141	0.145	0.151	0.145	0.145	0.145	0.150	0.140	0.140	0.170	0.028							
20	CP006647.2 *Borrelia miyamotoi* LB-2001	0.168	0.168	0.168	0.163	0.174	0.183	0.158	0.173	0.174	0.183	0.183	0.183	0.199	0.189	0.164	0.169	0.178	0.204						
21	KF422815.1 *Borrelia turcica* IST7	0.154	0.154	0.150	0.155	0.160	0.150	0.155	0.154	0.160	0.150	0.150	0.150	0.155	0.155	0.189	0.190	0.201	0.234	0.220					
22	CP002933.1 *Borrelia afzelii* PKo	0.149	0.149	0.149	0.144	0.163	0.178	0.163	0.173	0.163	0.178	0.178	0.178	0.173	0.183	0.178	0.194	0.199	0.215	0.230	0.194				
23	AE000783.1 *Borrelia burgdorferi* ss B31	0.178	0.178	0.178	0.173	0.194	0.183	0.194	0.204	0.194	0.189	0.183	0.189	0.178	0.189	0.214	0.231	0.225	0.242	0.252	0.209	0.058			

No.	Sequence	2	3	4	5	6	7	8	9	10	11	12	13	14	15	16	17	18	19	20	21	22	23	24	25
24	CP009117.1 *Borrelia valaisiana* Tom4006	0.164	0.164	0.168	0.163	0.194	0.189	0.173	0.183	0.173	0.189	0.189	0.189	0.173	0.194	0.194	0.215	0.215	0.231	0.231	0.188	0.063	0.067		
25	CP000013.1 *Borrelia bavariensis* PBi	0.183	0.183	0.183	0.178	0.209	0.209	0.189	0.199	0.189	0.209	0.209	0.199	0.204	0.215	0.220	0.242	0.231	0.247	0.264	0.220	0.089	0.089	0.072	
26	L42885.1 *Borrelia garinii* Ip90	0.174	0.174	0.173	0.168	0.189	0.204	0.178	0.189	0.178	0.204	0.204	0.194	0.199	0.209	0.209	0.209	0.236	0.253	0.247	0.199	0.071	0.085	0.063	0.024

*For a total of 252 positions. Blank spaces indicate complete identity.

Technical Appendix Table 4. Estimates of evolutionary divergence between *Borrelia* spp. *uvrA* sequences*

No.	Sequence	2	3	4	5	6	7	8	9	10	11	12	13	14	15	16	17	18	19
2	15–0335452																		
3	*Candidatus* Borrelia kalaharica M15–2747	0.002																	
4	CP011060.1 *Borrelia hermsii* CC1	0.088	0.086																
5	CP005829.1 *Borrelia anserina* BA2	0.111	0.108	0.100															
6	*Borrelia hispanica*	0.169	0.166	0.154	0.166														
7	gi|749302055 *Borrelia parkeri* HR1	0.112	0.109	0.091	0.128	0.159													
8	CP005851.1 *Borrelia parkeri* SLO	0.113	0.110	0.092	0.128	0.161	0.001												
9	CP000976.1 *Borrelia duttonii* Ly	0.171	0.168	0.155	0.160	0.046	0.156	0.157											
10	CP000993.1 *Borrelia recurrentis* A1	0.170	0.167	0.153	0.158	0.047	0.154	0.156	0.006										
11	gi|752506972 *Borrelia coriaceae* Co53	0.142	0.139	0.106	0.149	0.169	0.092	0.093	0.167	0.163									
12	gi|749307620 *Borrelia crocidurae* DOU	0.174	0.171	0.155	0.163	0.048	0.153	0.154	0.009	0.010	0.167								
13	CP003426.1 *Borrelia crocidurae* Achema	0.173	0.170	0.156	0.161	0.050	0.154	0.156	0.008	0.009	0.169	0.001							
14	CP000049.1 *Borrelia turicatae* 91E135	0.120	0.117	0.087	0.125	0.161	0.020	0.021	0.156	0.154	0.098	0.153	0.154						
15	CP009656.1 *Borrelia burgdorferi* sensu stricto B31	0.270	0.268	0.248	0.270	0.283	0.255	0.256	0.257	0.257	0.263	0.254	0.256	0.250					
16	CP009117.1 *Borrelia valaisiana* Tom4006	0.274	0.272	0.249	0.264	0.262	0.252	0.252	0.255	0.255	0.247	0.250	0.251	0.242	0.076				
17	CP002933.1 *Borrelia afzelii* PKo	0.262	0.260	0.249	0.269	0.268	0.248	0.250	0.261	0.261	0.255	0.256	0.258	0.245	0.082	0.070			
18	CP007564.1 *Borrelia garinii* SZ	0.279	0.277	0.257	0.273	0.277	0.257	0.258	0.259	0.258	0.267	0.256	0.258	0.253	0.077	0.061	0.066		
19	CP000013.1 *Borrelia bavariensis* PBi	0.272	0.270	0.257	0.267	0.270	0.253	0.255	0.254	0.253	0.265	0.251	0.253	0.245	0.081	0.059	0.067	0.017	

* Blank spaces indicate complete identity.

Technical Appendix Table 5. Estimates of evolutionary divergence in *Borrelia* spp. between 7 MLST gene sequences (*clpX, nifS, pepX, pyrG, recG, rplB, uvrA*)*

No.	Sequence	2	3	4	5	6	7	8	9	10	11	12
2	15–0335452											
3	*Borrelia anserina BA2*	0.101										
4	*Borrelia hermsii* DAH	0.110	0.114									
5	*Borrelia parkeri* SLO	0.112	0.124	0.088								
6	*Borrelia turicatae* 91E135	0.116	0.126	0.087	0.019							
7	*Borrelia coriaceae* Co53	0.140	0.146	0.111	0.102	0.101						
8	*Borrelia miyamotoi* LB2100	0.143	0.149	0.125	0.121	0.120	0.134					
9	*Borrelia crocidurae* Achema	0.173	0.166	0.147	0.148	0.148	0.160	0.170				
10	*Borrelia recurrentis* A1	0.173	0.165	0.147	0.147	0.146	0.159	0.168	0.006			
11	*Borrelia duttonii* Ly	0.174	0.165	0.148	0.147	0.146	0.160	0.169	0.007	0.003		
12	*Borrelia burgdorferi* B31	0.301	0.294	0.281	0.275	0.275	0.276	0.291	0.265	0.265	0.266	

*Blank spaces indicate complete identity.

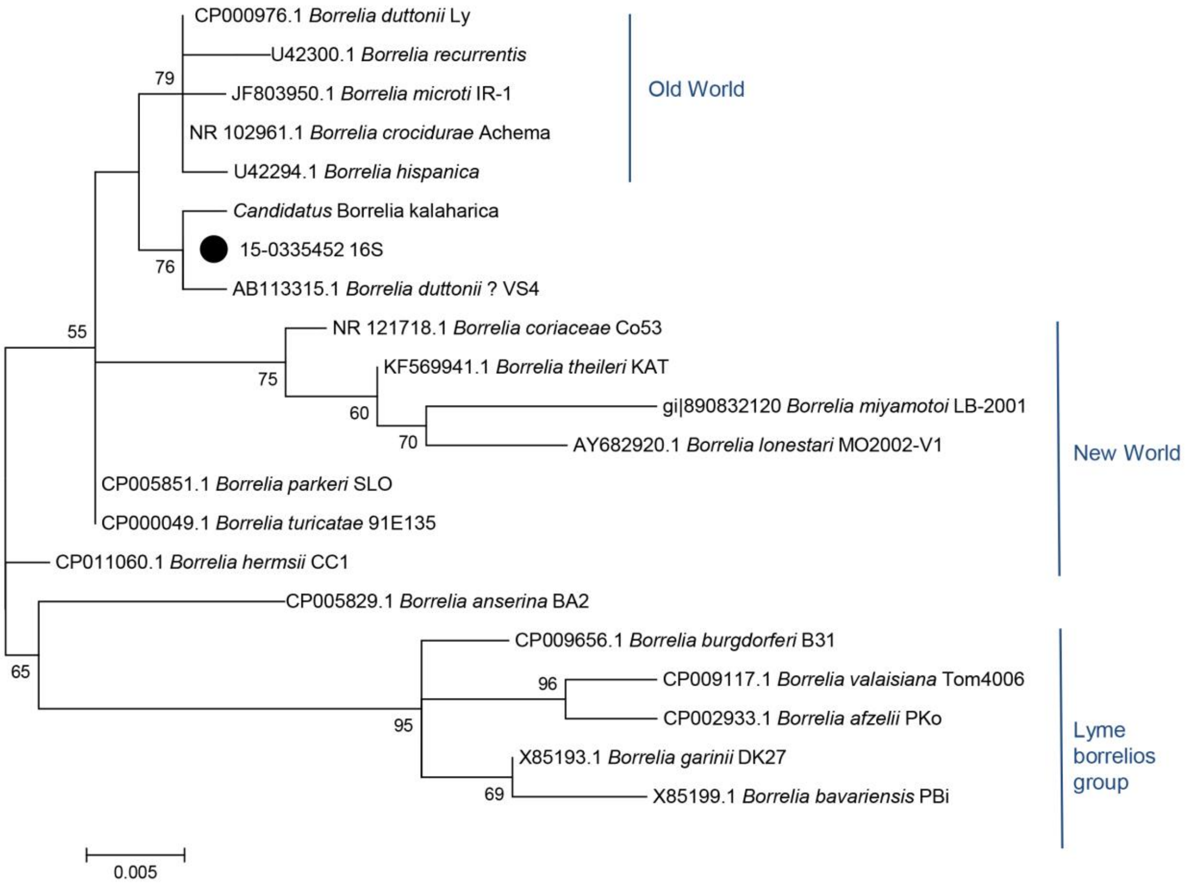

Technical Appendix Figure 1. Molecular phylogenetic analysis by maximum-likelihood method of the 16S locus. The tree with the highest log likelihood (–967.4651) is shown. A discrete gamma distribution was used to model evolutionary rate differences among sites (4 categories (+G, parameter = 0.0651)). The rate variation model allowed for some sites to be evolutionarily invariable ([+I], 50.1953% sites). Scale bar indicates substitutions per site. The analysis involved 21 nt sequences. There were 462 positions in the final dataset. Bootstrap values >50 are shown. Black dot indicates the sample analyzed in the present study.

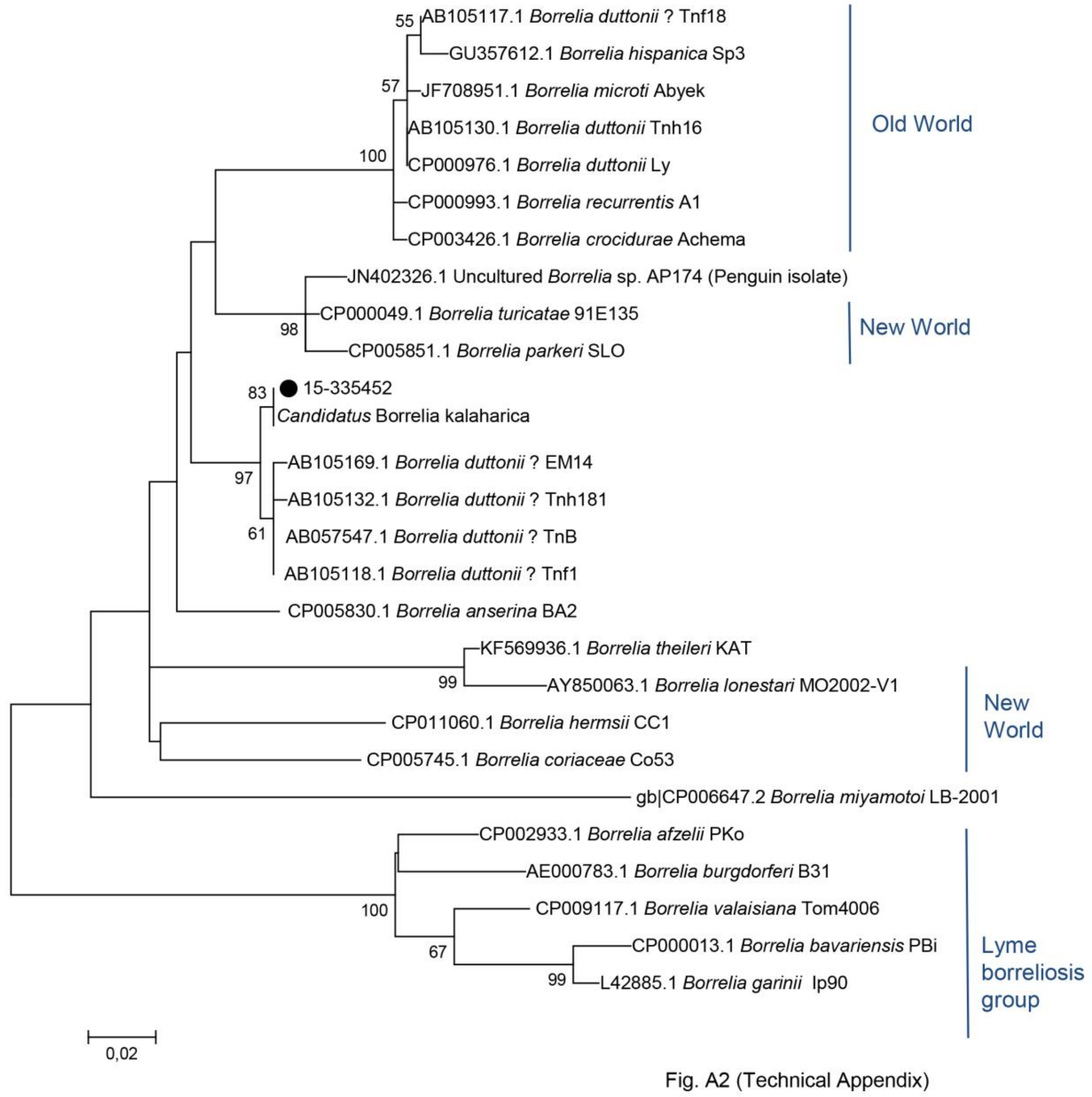

Technical Appendix Figure 2. Molecular phylogenetic analysis by maximum-likelihood method of *flaB* sequences. The tree with the highest log likelihood (–1352,7361) is shown. A discrete gamma distribution was used to model evolutionary rate differences among sites (4 categories (+G, parameter = 0,5498)). The rate variation model allowed for some sites to be evolutionarily invariable ([+I], 0,0000% sites). The analysis involved 27 nt sequences. There were 252 positions in the final dataset. Scale bar indicates substitutions per site. Bootstrap values >50 are shown. Black dot indicates the sample analyzed in the present study.
